# Inflammaging impairs peripheral nerve maintenance and regeneration

**DOI:** 10.1111/acel.12833

**Published:** 2018-08-31

**Authors:** Robert Büttner, Alexander Schulz, Michael Reuter, Asha K. Akula, Thomas Mindos, Annemarie Carlstedt, Lars B. Riecken, Stephan L. Baader, Reinhard Bauer, Helen Morrison

**Affiliations:** ^1^ Leibniz Institute on Aging Fritz Lipmann Institute Jena Germany; ^2^ Department of Genetics and Program in Cellular Neuroscience, Neurodegeneration and Repair Yale University School of Medicine New Haven Connecticut; ^3^ Institute of Anatomy University of Bonn Bonn Germany; ^4^ Institute of Molecular Cell Biology Jena University Hospital Jena Germany

**Keywords:** peripheral nervous system, inflammaging, neural regeneration, aging, macrophages, schwann cell

## Abstract

The regenerative capacity of peripheral nerves declines during aging, contributing to the development of neuropathies, limiting organism function. Changes in Schwann cells prompt failures in instructing maintenance and regeneration of aging nerves; molecular mechanisms of which have yet to be delineated. Here, we identified an altered inflammatory environment leading to a defective Schwann cell response, as an underlying mechanism of impaired nerve regeneration during aging. Chronic inflammation was detected in intact uninjured old nerves, characterized by increased macrophage infiltration and raised levels of monocyte chemoattractant protein 1 (MCP1) and CC chemokine ligand 11 (CCL11). Schwann cells in the old nerves appeared partially dedifferentiated, accompanied by an activated repair program independent of injury. Upon sciatic nerve injury, an initial delayed immune response was followed by a persistent hyperinflammatory state accompanied by a diminished repair process. As a contributing factor to nerve aging, we showed that CCL11 interfered with Schwann cell differentiation in vitro and in vivo*. *Our results indicate that increased infiltration of macrophages and inflammatory signals diminish regenerative capacity of aging nerves by altering Schwann cell behavior. The study identifies CCL11 as a promising target for anti‐inflammatory therapies aiming to improve nerve regeneration in old age.

## 1 INTRODUCTION

The mammalian peripheral nervous system (PNS) maintains a high regenerative capacity enabling long‐distance axon regeneration and substantial functional recovery, even in the adult (Fenrich & Gordon, 2004; Huebner & Strittmatter, 2009). This regenerative potential decreases in mammals of advanced age; peripheral nerve repair becomes slow, incomplete, and/or nonfunctional (Verdu, Ceballos, Vilches, & Navarro, 2000; Wang, Zhou, Shi, Smith, & Li, 2007). While this defect has long been described in humans and rodent model systems, progress in understanding molecular and cellular mechanisms underlying PNS aging is limited—hampering the development of rational rejuvenating therapies in aged patients. Therefore, we aimed to discover how aging impairs peripheral nerve maintenance and regeneration processes.

Following traumatic injuries, peripheral nerves undergo a multistep repair program of Wallerian degeneration, axonal regrowth, and target reinnervation. Hallmarks of Wallerian degeneration are as follows: (a) detachment of resident Schwann cells from associated axons, (b) transition of these Schwann cells into a “repair Schwann cell” phenotype, (c) breakdown of the blood–nerve barrier, and (d) influx of macrophages into the tissue that, (e) in concert with “repair Schwann cells,” phagocytize axonal and myelin‐derived debris (Chen, Yu, & Strickland, 2007; Jessen, Mirsky, & Lloyd, 2015). During the regeneration phase, macrophages support “repair Schwann cells” in mediating axonal regrowth to re‐innervate the target tissue (Cattin et al., 2015; Mietto, Mostacada, & Martinez, 2015; Mokarram, Merchant, Mukhatyar, Patel, & Bellamkonda, 2012). Regeneration is completed when inflammatory processes resolve and “repair Schwann cells” redifferentiate. Actions of several different cell types—neurons, Schwann cells, and immune cells—are required to ensure successful peripheral nerve repair.

In an interesting manner, the intrinsic growth capacity of neurons appears unaffected by aging (Kang & Lichtman, 2013), suggesting defects in older animals are due to an impaired environment with aged Schwann cells and macrophages being less effective at clearing debris. Two key studies have verified that the regenerating axonal environment is defective in old animals (Painter et al., 2014; Scheib & Hoke, 2016). The former observed age‐dependent differences in Schwann cell behavior and delayed repair program activation. The latter detected increased macrophage infiltration in old intact nerves, as well as an impaired immune response in vivo upon peripheral nerve injury in old age. Schwann cells and macrophages in vitro displayed an attenuated phagocytic activity, suggesting that slow nerve regeneration in old rodents is a failure of repair Schwann cell and macrophage function (Scheib & Hoke, 2016). However, details of cell‐intrinsic and cell‐extrinsic molecular pathways explaining abnormal Schwann cell repair responses are limited; the effect of the altered inflammatory environment on old (uninjured) nerves, as well as on the course of regeneration, remains unaddressed.

This study investigates the inflammatory nerve environment in intact and regenerating old nerves. We demonstrate an altered inflammatory nerve microenvironment as a contributing factor impairing peripheral nerve maintenance and regeneration in old age, by influencing Schwann cell repair processes.

## 2 RESULTS

### 2.1 Age‐related impairment of peripheral nerve regeneration

A growing body of work demonstrates age‐dependent decline of peripheral nerve regeneration capacity (Painter et al., 2014; Verdu et al., 2000; Wang et al., 2007), but insight into underlying mechanisms remains limited. To better understand age‐dependent factors impacting on peripheral nerve regeneration, we performed sciatic nerve crush injuries on C57BL/6 J mice of two different ages. Given the strain's average life expectancy of 24 months (Rowlatt, Chesterman, & Sheriff, 1976), we declared 20‐month‐old mice “old” and 6‐month‐old mice “mature adults” (Flurkey, Currer, & Harrison, 2007).

**Figure 1:**
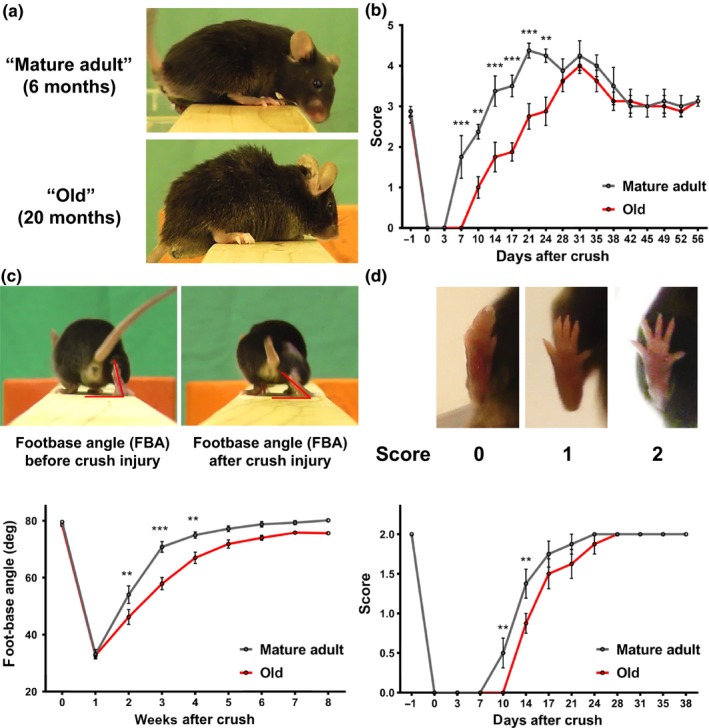
Aging impairs functional recovery after sciatic nerve crush injury. (a) “Mature adult” (6 months) and “old” (20 months) mice were subjected to sciatic nerve crush injury, and regeneration was assessed by monitoring recovery of sensory and motor functions. (b) Sensory recovery was tested by responsiveness of the paw to monofilaments of varying stiffness. Scoring reflects bending threshold forces: (0) no response for 300 g, (1) 300 g, (2) 4 g, (3) 2 g, (4) 0.4 g, and (5) 0.07 g. Motor recovery was tested by (c) measurement of the footbase angle and (d) toe spreading. Significances of all differences were calculated by two‐way ANOVA with Holm–Sidăk post hoc test and indicated by *p < 0.05, **p < 0.01, ***p < 0.001, mean ± *SEM*. *n* = 8 mice per age in b and d, *n* = 7 in c

Old mice show typical aging signs, such as kyphosis and shaggy fur (Figure 1a). Following sciatic nerve crush injury, these showed a significant delay in recovery of sensory functions, indicated by the Semmes–Weinstein monofilament test (Figure 1b). Most sensory recovery may have arisen from collateral sprouting, as the saphenous nerve remained uninjured and might have hyperinnervated the paw region, leading to the observed hypersensitivity (Duraku et al., 2012). We investigated recovery of motoric functions—measuring the footbase angle of mice in single‐frame motion analysis (SFMA) (Figure 1c) as a highly reproducible marker for functional muscle reinnervation (Fey, Schachner, & Irintchev, 2010). Again, old mice showed significantly delayed recovery of motoric functions, also exhibiting recovery delay in ability to spread their toes (Figure 1d)—an alternative marker for motor reinnervation efficacy after peripheral nerve damage (Ma et al., 2011). Our tests indicated delayed but almost full functional recovery of old mice after peripheral nerve crush injury.

**Figure 2:**
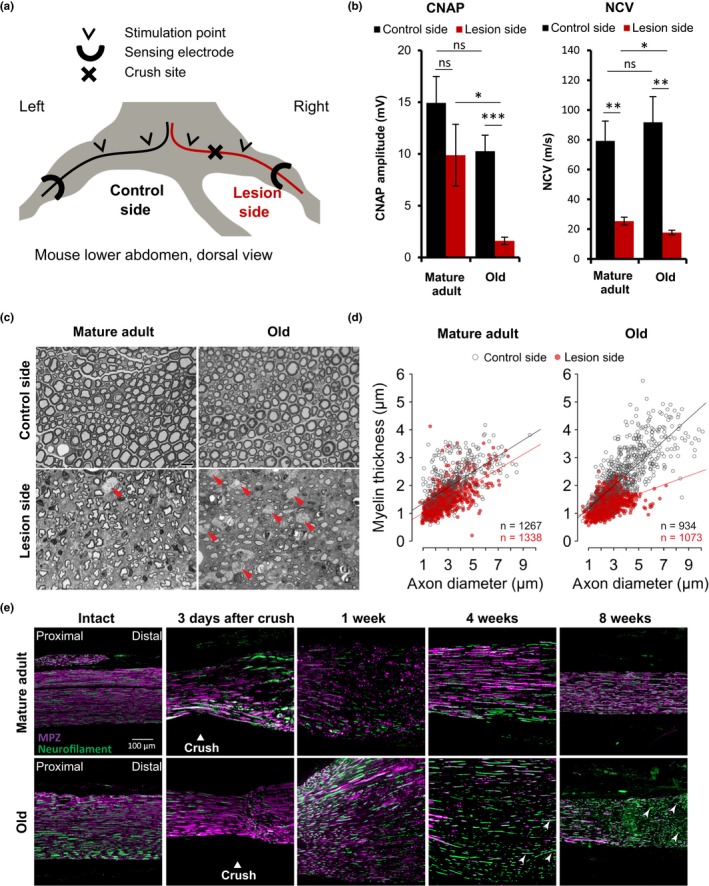
Aging impairs structural regeneration after sciatic nerve crush injury. (a) For electrophysiological measurements, mice were anesthetized and fixed in an illustrated set‐up 4 weeks after unilateral sciatic nerve crush injury. (b) Compound nerve action potentials (CNAP) and nerve conduction velocities (NCV) were measured in situ on crushed sciatic nerves and intact contralateral control nerves of six mature adult mice and eight old mice; mean ± *SEM*. *p < 0.05, ** *p* < 0.01, *** *p* < 0.001 by unpaired, two‐tailed *t*‐test. (c) Representative Toluidine blue‐stained semi‐thin cross‐sections of mature adult and old sciatic nerves 4 weeks after crush injury and intact contralateral, respectively. Cross‐sections in the crush area particularly of old sciatic nerves show multiple macrophages (red arrowheads); scale bar: 10 µm. (d) Myelin thickness relative to axon diameter was quantified in cross‐sections of uninjured (control side) and injured (lesion side) sciatic nerves of four mature adult and five old mice four weeks after crush injury and illustrated as scatter plot. Per nerve 156 to 448 axons plus myelin sheath were measured and the sum of quantified axons per age and side indicated within the plots. Linear mixed models with Tukey post hoc test indicate highly significant differences (*p* < 0.0001) between injured and uninjured nerves for both ages as well as between injured nerves of mature adult and old mice. (e) Timeline of representative longitudinal sciatic nerve sections at the crush site, before and at indicated time points after crush injury, immunostained for myelin protein zero (MPZ purple) to mark myelination and neurofilament (green) to mark axonal fibers; proximal left and distal right, white arrowheads point at unmyelinated axons in old mice far from the remyelination frontier, scale bar: 100 μm

Electrophysiological properties further reflect differences in functional nerve repair. Through in situ stimulation of the sciatic nerve proximal and distal of the crush site, we assessed compound nerve action potential (CNAP) and nerve conduction velocity (NCV) in intact and lesioned nerves from both cohorts (Figure 2a). Four weeks after crush injury, we saw a significantly lower CNAP in old mice compared to mature adults (Figure 2b), indicating a smaller number of functionally regenerated axons. Lesioned nerves of old mice exhibited a slower NCV (Figure 2b), suggesting reduced remyelination.

To assess nerve regeneration on a structural level, we analyzed intact control nerves and semi‐thin cross‐sections of sciatic nerves 4 weeks after crush injury (Figure 2c). Injured nerves of mature adult mice showed small axons with myelinated sheaths, most probable resembling remyelinated axons, and almost no macrophages; injured nerves of old mice displayed much less axons with smaller diameter and thin myelin sheaths and a high number of macrophages (red arrowheads). Quantification of myelin thickness relative to axon diameter revealed major differences between regenerating nerves in both groups, especially for larger axon diameters (Figure 2d). Investigation of axon density, average axon diameter, and myelin thickness thus revealed defects in regenerating sciatic nerves of old mice, whereas g‐ratio showed no age‐dependent decrease (Supporting information Figure S1). Similar results were obtained in immunohistochemical stainings of longitudinal sciatic nerve sections at different time points after injury (Figure 2e). Injured nerves of old mice showed delayed Wallerian degeneration three days after crush, followed by delayed and incomplete remyelination. Axonal regrowth was less affected by aging, indicated by axonal regrowth far distal to the remyelination frontier 4 and 8 weeks postinjury (white arrowheads, Figure 2e)—in contrast to the large drop in CNAP observed in sciatic nerves of old mice after injury (Figure 2b). This may be due to insufficient reinnervation as previously described and attributed to age‐related alterations in soluble target‐derived neurotrophic factors (Kovacic, Sketelj, & Bajrovic, 2009).

Our data suggest deficiencies in the morphological regeneration of aged peripheral nerves 4 weeks after nerve injury. We suppose Schwann cell functions—rather than axon‐intrinsic properties—undergo an age‐dependent decline and be causative for diminished peripheral nerve repair in old age.

### 2.2 Altered injury response and inflammatory microenvironment in old age

Wallerian degeneration is a prerequisite for efficient regeneration of injured nerves and involves several different cell types, including macrophages and other immune cells (Chen et al., 2007; Jessen et al., 2015).

**Figure 3:**
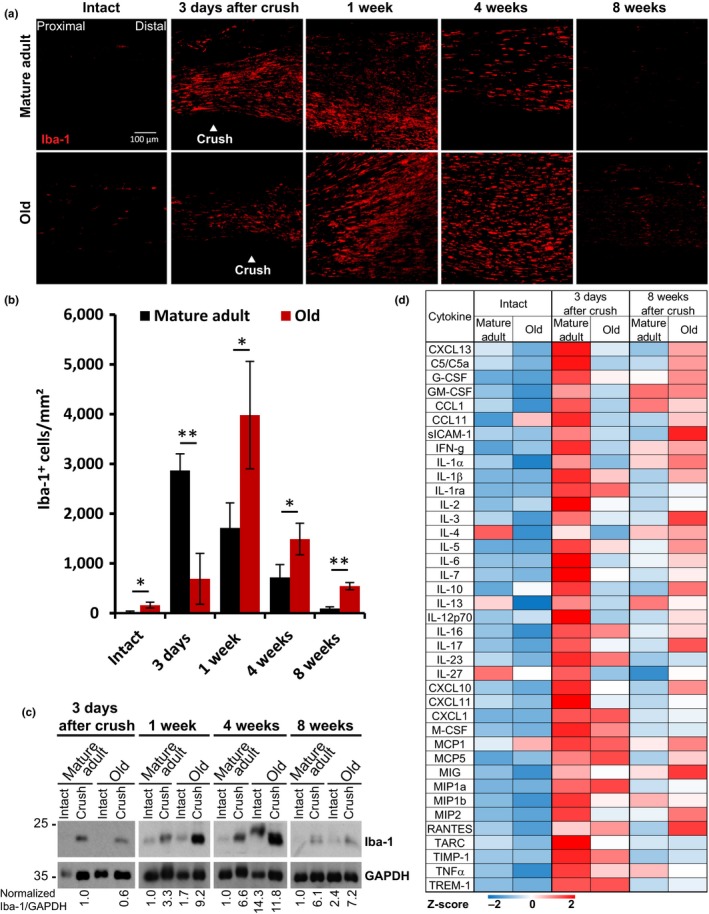
Age‐related changes of the nerve injury‐induced immune response. (a) Timeline of representative longitudinal sciatic nerve sections, before and at indicated time points after crush injury, immunostained for Iba‐1 to mark macrophages; scale bar: 100 μm. (b) Quantification of Iba‐1‐positive cells per area in immunostainings of *n* = 3 biological replicates; mean ±SD. * *p* < 0.05, ** *p* < 0.01 by unpaired, two‐tailed *t*‐test. (c) Immunoblots for Iba‐1 mark macrophage presence in lysates of intact or crushed sciatic nerves at indicated time points. Lysates were pooled from *n* = 3 different mice for all time points. The blot of 3 days after crush is from a different gel than the other samples. Equal loading is indicated by GAPDH. (d) Heatmap with row‐specific *Z*‐scores of a dot‐blot array (Supporting information Figure S2A) shows cytokine expression in intact and crushed sciatic nerve lysates. Pooled lysates from *n* = 3 mice per age and time point.

Previous work demonstrated age‐dependent changes to the immune system and its responses to injuries throughout different species and tissues (Montecino‐Rodriguez, Berent‐Maoz, & Dorshkind, 2013), yet the exact impact of age‐dependent immune system alterations on peripheral nerve maintenance and regeneration has barely been studied. We performed Iba‐1 immunostainings to identify macrophages in mature adult and old sciatic nerves at different time points before and after crush injury. The number of macrophages in intact sciatic nerves of old mice appeared to be increased, independent of injury, indicating a chronic inflammatory microenvironment within old nerves (Figure 3a,b). Compared to mature adults, old mice exhibited markedly reduced macrophage numbers soon after crush (3 days) but significantly overshooting macrophage infiltration in later phases (1–8 weeks after crush). This is consistent with the data shown in semi‐thin sections (Figure 2c). Iba‐1 immunoblots on sciatic nerve lysates confirmed this finding (Figure 3c), which indicated low‐grade chronic macrophage recruitment in peripheral nerves of old mice and delayed, but persisting injury‐induced hyperinflammatory response.

We dissected age‐related changes to the inflammatory microenvironment before and after crush injury, screening for various cytokines, chemokines, and acute‐phase proteins in nerve lysates of mature adult and old mice (Figure 3d and Supporting information Figure S2A). Age‐dependent changes in cytokine expression levels were detectable in both injured and intact nerves. Expression in mature adult mice was strongly elevated three days after injury, but efficiently downregulated eight weeks after. Cytokines in old mice showed lower activation 3 days after injury, but higher upregulation eight weeks after; old mice reveal delayed but prolonged cytokine expression, seemingly consistent with delayed but prolonged macrophage infiltration in old nerves (Figure 3b). In uninjured nerves, comparison of cytokine profiles identified age‐dependent downregulation of anti‐inflammatory cytokines interleukin 4 (IL‐4), IL‐13, and IL‐27, together with significant age‐dependent upregulation of the pro‐inflammatory cytokines monocyte chemoattractant protein 1 (MCP1) and CC chemokine ligand 11 (CCL11) (Figure 3d).

This deregulated inflammatory response to injury and the presence of a chronic low‐grade inflammatory environment in intact peripheral nerves of old mice, has been previously coined “inflammaging” in other tissues (Franceschi et al., 2007).

### 2.3 Anti‐inflammatory treatment strategy

**Figure 4:**
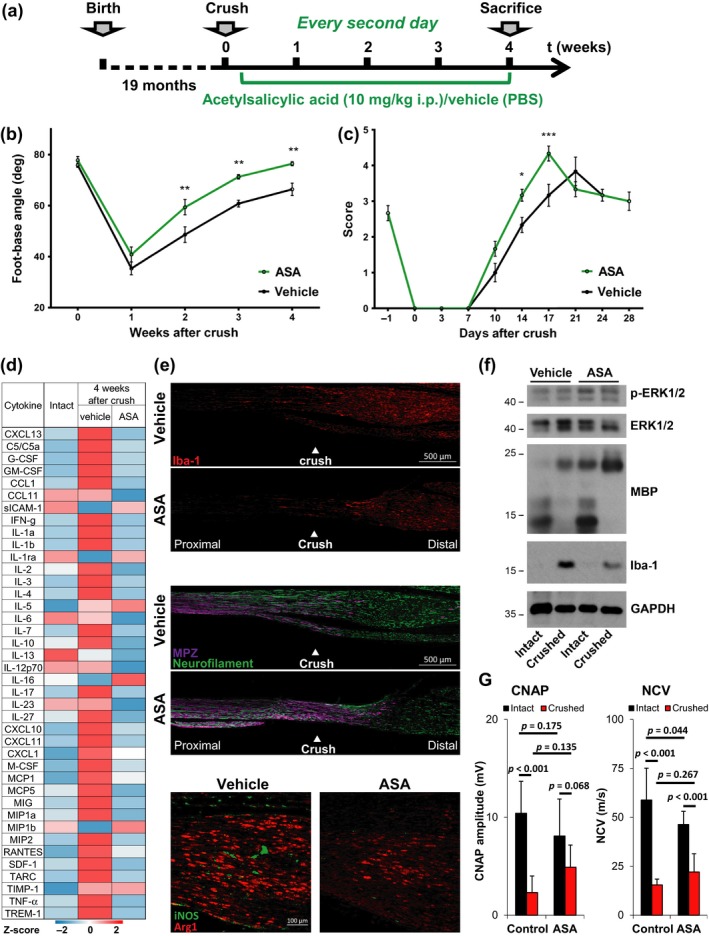
Acetylsalicylic acid improves peripheral nerve repair in old mice. (a) Two cohorts of *n* = 6 mice were subjected to unilateral sciatic nerve crush injury procedure and drug therapy with acetylsalicylic acid (ASA) or PBS (vehicle). ASA (10 mg per kg body weight) or PBS was injected intraperitoneally for four weeks, beginning on day 3 after crush injury and every second day thereafter. (b, c) Recovery of motor and sensory function was assessed using single‐frame motion analysis and Semmes–Weinstein monofilament test; *n* = 6 mice per group (*n* = 5 ASA‐treated mice in SFMA), significant differences determined in two‐way ANOVA with Holm–Sidăk post hoc test, *p < 0.05, **p < 0.01, mean ± *SEM*. (d) Heatmap with row‐specific Z‐scores of a dot‐blot array (Supporting information Figure S2C) to measure cytokine expression in pooled sciatic nerve lysates (*n* = 3 mice) of mature adult and old mice, treated with ASA or vehicle control. (e) Representative immunostainings of longitudinal sciatic nerve sections at the crush area of vehicle‐ and ASA‐treated mice 4 weeks after crush injury. Immunolabeling of Iba‐1, MPZ, and neurofilament indicate macrophage appearance, myelination, and axonal fibers; proximal left and distal right, scale bar: 500 μm. Immunostainings for Arginase1 and iNOS indicate M2 and M1 macrophage populations, proximal left and distal right, scale bar: 100 µm. (F) Immunoblot analysis of Erk1/2 expression and phosphorylation, MBP (myelinating Schwann cells) and Iba‐1 (macrophages). GAPDH indicates equal loading. Pooled sciatic nerve lysates from *n* = 3 mice. (G) Compound nerve action potentials (CNAP) and nerve conduction velocities (NCV) were measured in situ on crushed and intact sciatic nerves. *n* = 6 old mice treated with ASA or vehicle for 4 weeks after crush; mean ± *SD*. p‐values were calculated by two‐way ANOVA with Holm–Sidăk post hoc test and indicated in the diagrams

Acetylsalicylic acid (ASA) inhibits mammalian innate immune responses (Morris et al., 2009) and decreases macrophage infiltration in sciatic nerves (Schulz et al., 2016). To test whether suppression of the injury‐induced, hyperinflammatory response in old mice by ASA could improve peripheral nerve regeneration, we set up a four‐week treatment protocol using two cohorts of old mice (Figure 4a). “ASA” animals received a low dose of ASA (10 mg/kg in PBS) every second day, starting day 3 after injury. “Vehicle” control animals received equivalent volumes of PBS only. Efficacy of treatment was tested by monitoring recovery of motoric functions using SFMA (Figure 4b) and toe‐spread analysis (Supporting information Figure S2B), and sensoric functions using Semmes–Weinstein monofilament test (Figure 4c). ASA treatment had significant beneficial effects on all tested parameters.

Cytokine profiling confirmed the efficacy of ASA treatment in suppressing the persistent inflammatory response in old mice four weeks after injury (Figure 4d and Supporting information Figure S2C). Cytokines were downregulated to uninjured control levels or below, including MCP1 and CCL11. The impact of ASA on macrophage infiltration was evaluated four weeks after crush injury by stainings of longitudinal sections (Figure 4e). Macrophages in general were stained by Iba‐1, pro‐inflammatory M1 and proregenerative M2 macrophages were discriminated by iNOS and Arginase1. Quantification of the stainings (Supporting information Figure S2D) revealed a significant reduction in (a) overall cell density (stained by DAPI), (b) total macrophage numbers (Iba‐1), (c) pro‐inflammatory M1 macrophages (iNOS), and (d) proregenerative M2 macrophages.

The reduced pro‐inflammatory response was accompanied by improved remyelination, indicated by increased myelin protein zero (MPZ) signal in tissue sections (Figure 4e) and strong upregulation of the remyelination‐specific 21.5‐kDa isoform of myelin basic protein (MBP) (Harauz & Boggs, 2013) in whole nerve lysates (Figure 4f). Phosho‐ERK1/2 was slightly increased following ASA treatment, again indicating improved regeneration. Also, electrophysiological measurements revealed increased CNAP and NCV in ASA‐treated old mice four weeks after crush, but this trend did not reach statistically significant levels (Figure 4g).

Our data demonstrate significant reduction in injury‐induced inflammatory responses following low‐dose ASA treatment and highlight the beneficial effects of anti‐inflammatory treatment on peripheral nerve regeneration in old mice.

### 2.4 Age‐dependent alterations of the intact peripheral nerve

**Figure 5:**
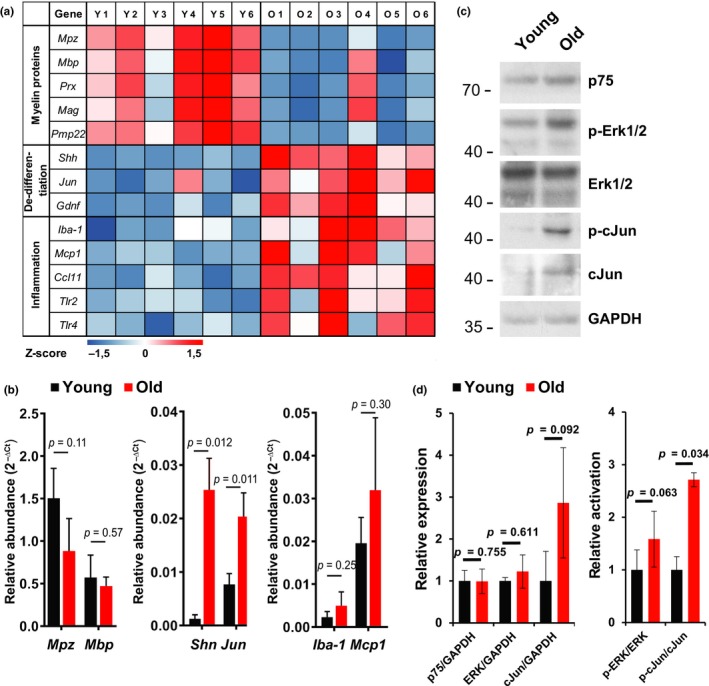
Inflammaging in sciatic nerves of old mice correlates with insufficient repair. (a) Expression of selected genes involved in myelination, dedifferentiation, and inflammation quantified from intact sciatic nerves of *n* = 6 young (3 months) and old (20 months) mice by RNA‐Seq. Obtained total read counts are illustrated in a heatmap with row‐specific Z‐scores. (b) Based on RNA‐Seq data, selected marker genes for myelination (*Mpz, Mbp*), dedifferentiation (*Shh, Jun*), and inflammation (*Iba-1, Mcp1*) were further validated by qPCR. *n* = 3 biological replicates for each gene and age except Shh young, where *n* = 2; mean ± *SD* of relative abundance. Indicated p‐values calculated by unpaired, two‐tailed *t*‐test. (c) Immunoblots of p75, Erk1/2, and cJun protein expression and phosphorylation, that is, activation of Erk1/2 and cJun, which are involved in Schwann cell repair program control. Equal loading indicated by GAPDH. Pooled sciatic nerve lysates from *n* = 3 mice. (d) Quantification of relative expression and phosphorylation, that is, activation, of Erk and cJun. *n* = 3 biological replicates. Significant differences between means ±SEM calculated by unpaired, two‐tailed *t*‐test, *p*‐values indicated in the diagrams

Inflammaging in intact old nerves correlated with an altered cytokine profile. We expected these intact aged nerves to be altered and primed for regeneration deficits. Thus, we compared the transcriptome of six intact sciatic nerves each from young (3 months) and old mice (20 months). Analysis of 26,840 genes identified 2,323 differentially expressed genes (DEGs) (1,230 upregulated in old; 1,093 upregulated in young mice). Using DEGs of young and old mice, we performed a PANTHER Enrichment analysis for biological processes, with Bonferroni correction for multiple testing. The ten most enriched Gene Ontology (GO) groups indicated for young mice, in principle, lipid synthesis processes (Supporting information Figure S3A) and for old mice, predominantly, activation of the immune system (Supporting information Figure S3B). We focused our analysis of the RNA‐Seq data on selected DEGs involved in either myelination, dedifferentiation, or inflammation (Figure 5a). While the selected myelination‐associated genes (*Mpz, Mbp, Prx, Mag, Pmp22*) were consistently higher expressed in younger mice, investigated genes involved in Schwann cell dedifferentiation (*Shh, Jun, Gdnf*) were more abundant in intact nerves of old mice. Old nerves also showed higher expression of macrophage markers Iba‐1 and Toll‐like receptors 2 and 4 (*Tlr2, Tlr4*); both said to be highly involved in Wallerian degeneration (Boivin et al., 2007). These data confirmed our enrichment analysis that inflammaging accompanies reduced myelination in intact sciatic nerves of old mice. Further validation by qPCR confirmed (Figure 5b) our results. Of note, both candidates identified in the cytokine array—MCP1 and CCL11—showed significantly elevated gene expression (*MCP1, Ccl11*) in old age, and investigation of CCL11‐receptors (CCR2, CCR3, CCR5) revealed CCR5 gene expression significantly upregulated with age (Supporting information Figure S3C). Further, MCP1 and CCL11 gene expressions were strongly upregulated after dissection in an explant study (Supporting information Figure S3D), indicating an important role for “inflammaging” in peripheral nerves.

To better understand the reasons for diminished myelination in aged peripheral nerves, we screened for alterations of intrinsic Schwann cell repair pathways such as the Ras/Raf/ERK and the cJun (Arthur‐Farraj et al., 2012; Harrisingh et al., 2004; Napoli et al., 2012) in intact sciatic nerves. Immunoblot analysis (Figure 5c, d) revealed no changes of p75 or Erk1/2 protein expression, but Erk1/2 protein appeared more phosphorylated in old animals. Old mice also showed increased cJun expression and phosphorylation. These pathway changes may indicate a persistent repair attempt in intact old nerves (Napoli et al., 2012; Parkinson et al., 2008).

In longitudinal nerve tissue sections, the increased phospho‐Erk1/2 and cJun signal was partially colocalized and clustered with p75*, *a marker for immature and “repair” Schwann cells (Jessen & Mirsky, 2008) (Supporting information Figure S4). Thus, the inflammatory microenvironment in aged peripheral nerves appears to correlate with persistent low‐level, yet insufficient, repair processes. Some non‐nuclear cJun was also colocalized with the macrophages marker F4/80 in old nerves, indicating phagocytic activity. However, a proportion of Schwann cells persists in old intact peripheral nerves in an undifferentiated state, seemingly incapable of proper myelination—some of which may represent denervated Schwann cells remaining after age‐dependent axonal degeneration.

Our data suggest that some Schwann cells in old peripheral nerves are in constant nonfunctional repair mode, independent of injury. Whether inflammaging induces this cellular response, or Schwann cells are critical for inducing chronic inflammation, is unclear.

### 2.5 CCL11 attenuates schwann cell myelination in vitro and in vivo

To elucidate the connection between inflammaging and diminished remyelination in old peripheral nerves, we maintained focus on CCL11 and MCP1, which were upregulated with age in our cytokine profiling and RNA‐Sequencing approaches (Figures 3d and 5a). MCP1 as a potent macrophage attracting factor is known to be expressed by denervated Schwann cells (Deshmane, Kremlev, Amini, & Sawaya, 2009; Tofaris, Patterson, Jessen, & Mirsky, 2002). CCL11, also known as eotaxin‐1, has been identified as chemoattractant for eosinophile immune cells (Jose et al., 1994) and is stated to be secreted by M1 and M2 macrophages (Arango Duque & Descoteaux, 2014; Herranz, Traves, Luque, & Hortelano, 2012). Both cytokines have been found locally expressed in sciatic nerves within two days after injury (van Rossum, Hilbert, Strassenburg, Hanisch, & Bruck, 2008). We confirmed their local upregulation upon injury by explant cultures (Supporting information Figure S3D), supporting a crucial role in normal peripheral nerve repair, as well as inflammaging. While high levels of MCP1 are likely causal for increased macrophage infiltration of old intact sciatic nerves, the impact of CCL11 on aged peripheral nerves remains obscure. CCL11 binds to CC chemokine receptor (CCR) types 2, 3, and 5. Yet, expression of CCR3—the main receptor implicated in eosinophile attraction (Gao et al., 1996)—was barely detectable in our transcriptome analysis, while CCR2 and CCR5 were significantly expressed. Moreover, similar to its ligand CCL11, CCR5 was upregulated in old age (Supporting information Figure S3C). CCR5 has been found expressed by both Schwann cells and macrophages, with significant upregulation upon peripheral nerve injury (Kiguchi, Maeda, Kobayashi, Fukazawa, & Kishioka, 2010). We hypothesized that CCL11 may be directly involved in regulation of Schwann cell behavior and tested this in a coculture system with DRG neurons and Schwann cells.

**Figure 6:**
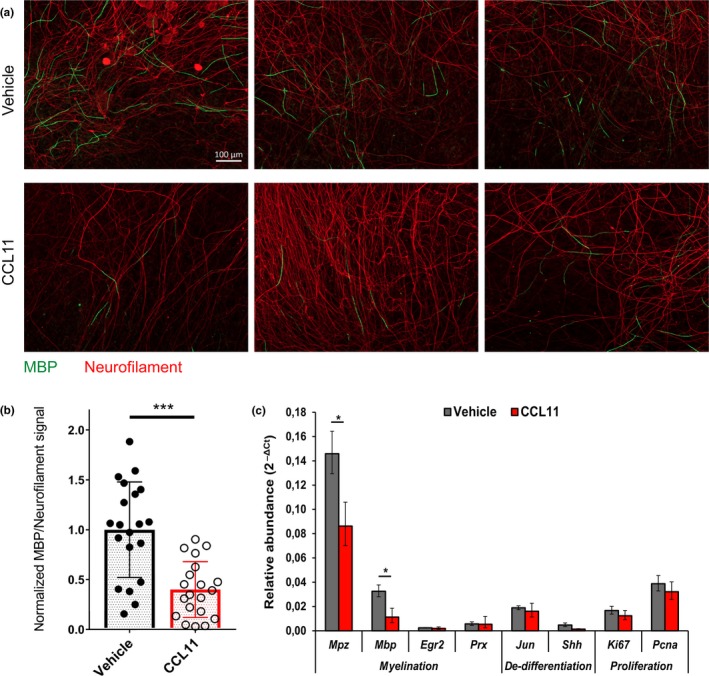
CCL11 impairs Schwann cell myelination in DRG coculture. DRG‐derived neurons and Schwann cells were cocultured for 6 days in normal growth medium plus 8 days in myelination‐promoting medium, both containing CCL11 (100 ng/ml) or vehicle (0.1% BSA) in PBS. (a) Representative pictures of CCL11‐ and vehicle‐treated cocultures after myelination, stained for myelin basic protein (MBP, green) and neurofilament protein H (neurofilament, red), scale bar: 100 µm. (b) Quantification of induced myelination per neurons in CCL11‐ and vehicle‐treated cocultures by normalized ratio between MBP and neurofilament signal. *n* = 10 biological replicates (DRG explants) quantified per experiment in two independent experiments. Scatter plot diagram of mean ± *SD* of normalized ratios between MBP and neurofilament signal. ***p < 0.001 by unpaired, two‐tailed *t*‐test. (c) Representative quantitative qPCR for two independent experiments measuring myelination, dedifferentiation, and proliferation markers in CCL11‐ and vehicle‐treated cocultures following incubation in myelination‐promoting medium. *n* = 4 biological replicates; mean ± *SD*. *p < 0.05 by unpaired, two‐tailed *t*‐test

Dissected DRGs from mouse embryos (E13.5) were cultivated for six days in cultivation medium, followed by eight days in myelination medium containing CCL11 or vehicle. Myelination was evaluated by stainings and qPCR (Figure 6). Staining for MBP as myelination marker and neurofilament heavy polypeptide as neuronal marker revealed significantly less myelin sheaths per axons in CCL11‐treated samples (Figure 6a,b). qPCR analysis showed significantly lower expression of the myelin markers *MPZ *and *Mbp *for CCL11‐treated cocultures. Other myelin markers, and markers for dedifferentiation or proliferation, were unchanged, indicating a specific role of CCL11 for myelination.

**Figure 7:**
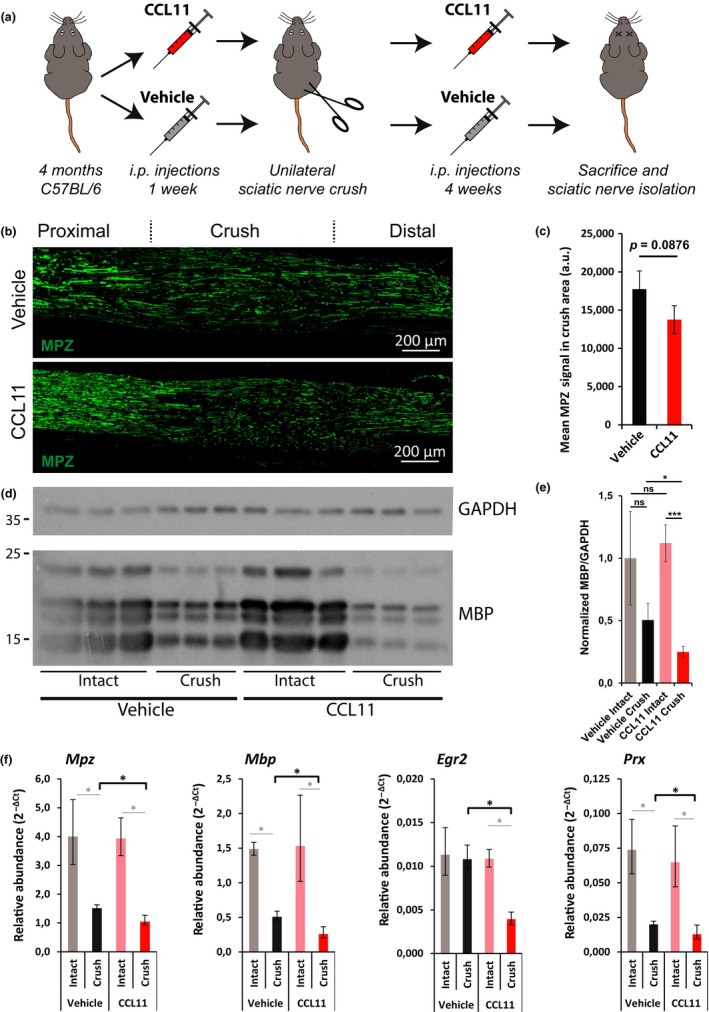
Decreased sciatic nerve remyelination in CCL11‐treated mice. (a) Scheme of in vivo experiment. One week before and four weeks after unilateral sciatic nerve crush injury, CCL11 (10 µg/kg body weight in PBS) or vehicle (PBS) was injected intraperitoneally every third to fourth day. Four weeks after crush injury, mice were sacrificed and sciatic nerves isolated. (b) Representative longitudinal sciatic nerve sections of vehicle‐ and CCL11‐treated mice four weeks after crush injury stained for myelin protein zero (MPZ, green) as marker for remyelination; crush area centered, proximal left, distal right, scale bar: 200 µm. (c) Quantification of mean MPZ signal in the crush area. *n* = 3 biological replicates per cohort; mean ± *SD*. *p*‐value calculated by unpaired, two‐tailed *t*‐test. (d) Immunoblots of MBP and GAPDH in crushed and intact sciatic nerves of *n* = 3 vehicle‐ and CCL11‐treated mice four weeks after injury. (e) Quantification of D. *n* = 3 biological replicates; mean ± *SD*. *p < 0.05, ***p < 0.001 with unpaired, two‐tailed *t*‐test. (f) Quantification of myelin protein genes expression by qPCR. *n* = 3 biological replicates; mean ± SD. *p < 0.05 with unpaired, two‐tailed *t*‐test

To evaluate the effect of CCL11 on Schwann cell behavior in vivo (Figure 7), we continuously injected CCL11 or vehicle (PBS) to cohorts of mature adult mice, starting 1 week before and ending 4 weeks after unilateral sciatic nerve crush injury; remyelination of regenerated and contralateral intact nerves was also evaluated (Figure 7a). We did not observe an altered macrophage infiltration behavior upon CCL11 injections (data not shown). However, MPZ signal intensity at the crush area tended toward less remyelination in CCL11‐treated mice (Figure 7b,c). Analysis of remyelination by myelin basic protein (MBP) immunoblot showed significantly reduced expression in CCL11‐treated mice, indicating reduced remyelination (Figure 7d,e). qPCR analysis illustrated significant reductions in myelination marker mRNAs (*Mpz, Mbp, Egr2, Prx*) in crushed sciatic nerves of CCL11‐treated versus vehicle‐treated mice (Figure 7f). Unlike injured nerves, we saw no significant differences between intact sciatic nerves of either group (data not shown), pointing to a prominent effect of CCL11 particularly on remyelination.

Our data demonstrate chronically elevated CCL11 expression in aged peripheral nerves and provide in vitro and in vivo evidence that CCL11 interferes with Schwann cell remyelination.

## 3 DISCUSSION

Age‐dependent decline of peripheral nerve regenerative capacities has previously been reported, yet underlying mechanisms remain poorly understood. Engaging several methods, we set out to comprehensively describe age‐dependent changes on functional, structural, cellular, and molecular levels. Our experimental design confirmed and further detailed previous work in this area (Figures 1 and 2) (He, Yadgarov, Sharif, & McCluskey, 2012; Painter et al., 2014; Scheib & Hoke, 2016; Verdu et al., 2000).

We demonstrated that age‐dependent regenerative impairments are associated with delayed, but also persistent hyperinflammatory response (Figure 3). Delayed immune responses were previously deemed culpable for poor peripheral nerve regeneration in old age (Scheib & Hoke, 2016). We show that, after an initial delay, injury‐induced immune responses are dramatically upregulated, resulting in a persistent, hyperinflammatory state even eight weeks postinjury—proven by continuing macrophage presence and inflammatory cytokine expression (Figure 3). Therefore, we suggest this persistent inflammatory state in old mice hinders efficient nerve regeneration—described as “inflammaging” in other tissues (Franceschi et al., 2007).

Pro‐inflammatory populations of macrophages significantly suppress peripheral nerve repair (Mokarram et al., 2012), pertinent to our suggestion that the hyperinflammatory environment is a major inhibitory factor of nerve recovery in old mice. Acetylsalicylic acid (ASA), shown to decrease the macrophage number in sciatic nerves (Schulz et al., 2016), was the drug chosen to assess an anti‐inflammatory therapy for old mice subjected to peripheral nerve injury—reasoning that repressing injury‐induced abnormal hyperinflammatory responses should augment nerve recovery in old mice. Following crush injury, accelerated functional recovery, accompanied by advanced remyelination and decreased macrophage appearance, was observed in ASA‐treated mice (Figure 4). Assessment of the motor and sensory recovery confirmed the beneficial effects of ASA treatment in old mice. Thus, we support anti‐inflammatory drug therapy in the context of peripheral nerve repair, especially for the elderly.

Age‐dependent inflammatory changes to the nerve microenvironment are detectable in uninjured old nerves and characterized by chronic macrophage infiltration, elevated cytokine expression and gene expression of pro‐inflammatory markers (Figures 3 and 5). Such low‐grade innate immune activation and inflammatory shift of the microenvironment, coined “inflammaging” (Franceschi et al., 2007), has been reported for several different organ systems (Shaw, Joshi, Greenwood, Panda, & Lord, 2010). Its impact on peripheral nerve maintenance and regeneration remains unknown, thus precluding mechanistic investigation.

Our study identified two key cytokines—MCP1 and CCL11—significantly upregulated in old uninjured sciatic nerves (Figure 3d and 5a). Expression of MCP1 is reported for denervated Schwann cells, to recruit macrophages to the injury site (Deshmane et al., 2009; Tofaris et al., 2002), then assist Schwann cells in cellular debris clearing, and stimulation of axonal regrowth (Dubovy, Jancalek, & Kubek, 2013; Jessen et al., 2015). But persistent MCP1 expression produces a chronic inflammatory state, hindering nerve regeneration (Kato et al., 2001). We suggest that the elevated MCP1 detected in old nerves is likely derived from undifferentiated Schwann cells and is causal for the observed increase in macrophage infiltration. In contrast, CCL11 did not exhibit an effect on macrophage behavior. But CCL11 is expressed by M1 and M2 macrophages (Arango Duque & Descoteaux, 2014; Herranz et al., 2012). Our sciatic nerve explant cultures confirmed that CCL11 expression is normally upregulated after nerve injury (Supporting information Figure S3D) (van Rossum et al., 2008). Young and old peripheral nerves secrete CCL11 in the context of regeneration, but levels are already chronically elevated in old peripheral nerves independent of injury. Elevated CCL11 serum levels are detected in mice and humans of old age, negatively regulating neurogenesis in the CNS (Villeda et al., 2011) and interfering with nervous system functions. We demonstrated that CCL11 directly interferes with Schwann cell myelination in vitro (Figure 6) and in vivo (Figure 7)—suggesting it as an important component of the dysregulated inflammatory nerve microenvironment impairing peripheral nerve remyelination in old age; a notion supported by age‐dependent increases in CCR5 expression (Supporting information Figure S3C), a CCL11 receptor reported to be expressed by Schwann cells and upregulated upon peripheral nerve injury (Kiguchi et al., 2010). Thus, elevated systemic CCL11 levels may be partly responsible for Schwann cell dedifferentiation and injury‐independent repair program activation (Figure 5) in intact old nerves. Of note is that at least some of the dedifferentiated Schwann cells in old nerves are likely derived from denervated Schwann cells following age‐dependent axonal degeneration. These cells could also be a source of CCL11 (Supporting information Figure S3D) and thus affect additional Schwann cells.

So, injury‐independent chronic CCL11 presence in intact nerve appears to prime Schwann cells into a constant dedifferentiated nonfunctional repair mode, impairing peripheral nerve maintenance. Further, injury‐induced CCL11 presence likely impairs Schwann cell repair activity and maturation during regeneration. We have now identified CCL11 as an important age‐dependent pro‐inflammatory circulating factor, representing a promising therapeutic target for improved peripheral nerve maintenance and repair in the elderly.

## 4 EXPERIMENTAL PROCEDURES

### 4.1 Experimental animals

All animal procedures were approved by the local authorities (Thüringer Landesamt für Verbraucherschutz, Germany) and conformed to international guidelines on ethical use of animals. All animals were on a C57BL/6 J background. For housing conditions see Supporting Information Appendix S1.

### 4.2 Sciatic nerve crush injury

Unilateral injuries of sciatic nerves were performed with minimal invasion, as described previously (Schulz et al., 2016). For details see Supporting Information Appendix S1.

### 4.3 Drug treatment

Application of ASA (Sigma‐Aldrich, St. Louis, MO, USA), 10 mg per kg body weight, was performed as previously described (Schulz et al., 2016). Recombinant murine Eotaxin/CCL11 (PeproTech, Hamburg, Germany) was injected intraperitoneally at 10 µg per kg body weight from one week before until four weeks after crush injury. For details see Supporting Information Appendix S1.

### 4.4 Assessment of motor and sensory recovery

The description of single‐frame motion analysis (SFMA) can be found in Fey et al. (2010), toe‐spread test was performed as previously described in Ma et al. (2011) and Semmes–Weinstein monofilament test was conducted according to Bradman, Ferrini, Salio, and Merighi (2015). For details see Supporting Information Appendix S1.

### 4.5 Electrophysiology

Sciatic nerve conduction characteristics were measured as described in Schulz, Walther, Morrison, and Bauer (2014) and detailed in Supporting Information Appendix S1.

### 4.6 Immunohistochemistry

Paraffin‐embedded sections of sciatic nerves were processed as described in Supporting Information.

### 4.7 Morphometric and ultrastructural analysis

Analysis of axon density, average axon diameter, and myelination thickness was conducted on semi‐thin sections of sciatic nerves, isolated from transcardially perfused mice. For details see Supporting Information Appendix S1.

### 4.8 Nerve lysis

Sciatic nerves of three different mice were pooled and flash frozen in liquid nitrogen immediately after isolation. Nerves were homogenized in a Precellys^® ^24 homogenizer (Bertin Instruments, Montigny‐le‐Bretonneux, France) in Pierce RIPA buffer (Thermo Fisher Scientific Inc., Waltham, MA, USA) with cOmplete protease inhibitor and phosSTOP phosphatase inhibitor (Roche Diagnostics GmbH, Mannheim, Germany).

### 4.9 Immunoblotting

For details see Supporting Information Appendix S1.

### 4.10 Cytokine detection

150 μg pooled nerve lysate was applied on Mouse Cytokine Array Panel A (R&D Systems, Minneapolis, MN, USA). Signals of dot‐blots were analyzed by pixel density quantification (ImageJ v1.47t). Cytokine‐specific changes between cohorts were visualized by row‐specific Z‐scores in a heatmap.

### 4.11 RNA‐Seq

For details see Supporting Information Appendix S1.

### 4.12 DRG cocultures

For details see Supporting Information Appendix S1.

### 4.13 QPCR

RNA was isolated and cDNA transcribed with EvoScript Universal cDNA Master (Roche Diagnostics GmbH, Mannheim, Germany). Gene expression was analyzed with A600A Go Taq^® ^qPCR Master Mix (Promega, Madison, WI, USA) in a LightCycler^® ^480 (Roche Diagnostics GmbH, Mannheim, Germany). For details see Supporting Information Appendix S1.

### 4.14 Statistical analysis

For details see Supporting Information Appendix S1.

## CONFLICT OF INTEREST

The authors declare no conflict of interest.

## AUTHOR CONTRIBUTIONS

RB, AS, and HM conceived and designed the study. RB performed and analyzed most experiments and prepared the manuscript. HM and AS supervised the experimental program. HM, AS, and LBR edited the manuscript. AC and TM performed some immunohistochemistry and immunoblotting. MR contributed to cytokine analysis, qPCR, and mouse work. MR and TM contributed to RNA‐seq. AKA performed most DRG coculture work. SLB performed morphometric analysis of nerves. R Bauer analyzed the electrophysiology. All authors approved the final version of the manuscript.
