# Supplementary material for: Inflammaging impairs peripheral nerve maintenance and regeneration
Source: Aging Cell. 2018 Aug 31;17(6):e12833. doi: 10.1111/acel.12833 (PMC6260910; doi:10.1111/acel.12833)
Supplement: Supplementary file 1 [file ACEL-17-e12833-s001.pdf]

Supporting Information

Inflammaging impairs peripheral nerve maintenance and regeneration

Robert Büttner, Alexander Schulz, Michael Reuter, Asha K. Akula, Thomas Mindos, Annemarie Carlstedt, Lars B. Riecken, Stephan L. Baader, Reinhard Bauer, Helen Morrison

Supporting Information Inventory

Figure S1, related to Figure 2 page 2

Figure S2, related to Figure 3 & 4 page 3

Figure S3, related to Figure 5 page 4

Figure S4, related to Figure 5 page 5

Supporting figures legends page 6

Supporting experimental procedures page 9

Figure S1

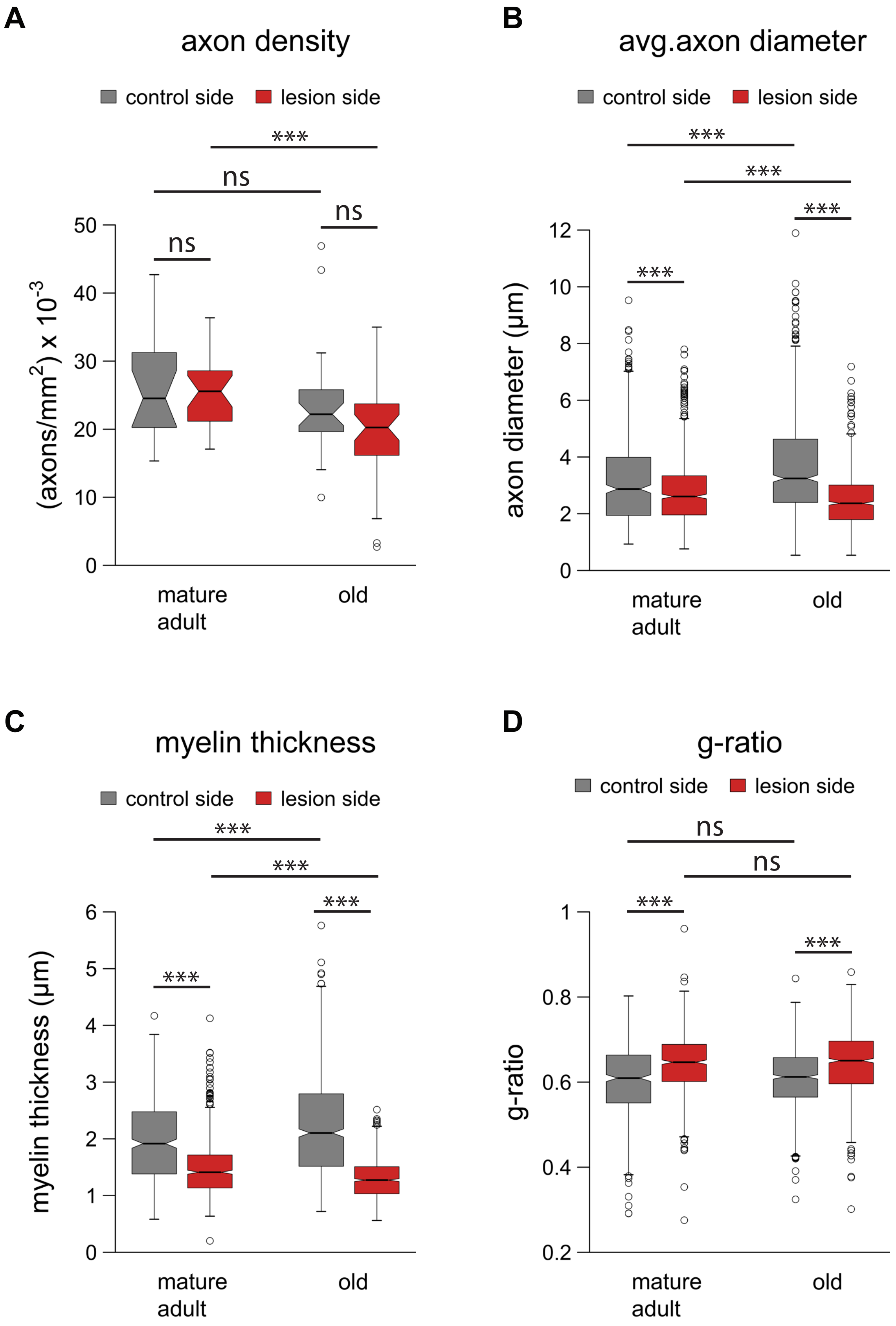
A
axon density
control side
lesion side
***
ns
ns
ns
50
40
30
20
10
0
(axons/mm2) x 10-3
mature adult
old
B
avg.axon diameter
control side
lesion side
***
***
***
***
12
10
8
6
4
2
0
axon diameter (µm)
mature adult
old
C
myelin thickness
control side
lesion side
***
***
***
***
6
5
4
3
2
1
0
myelin thickness (µm)
mature adult
old
D
g-ratio
control side
lesion side
ns
ns
***
***
1
0.8
0.6
0.4
0.2
g-ratio
mature adult
old

Figure S2

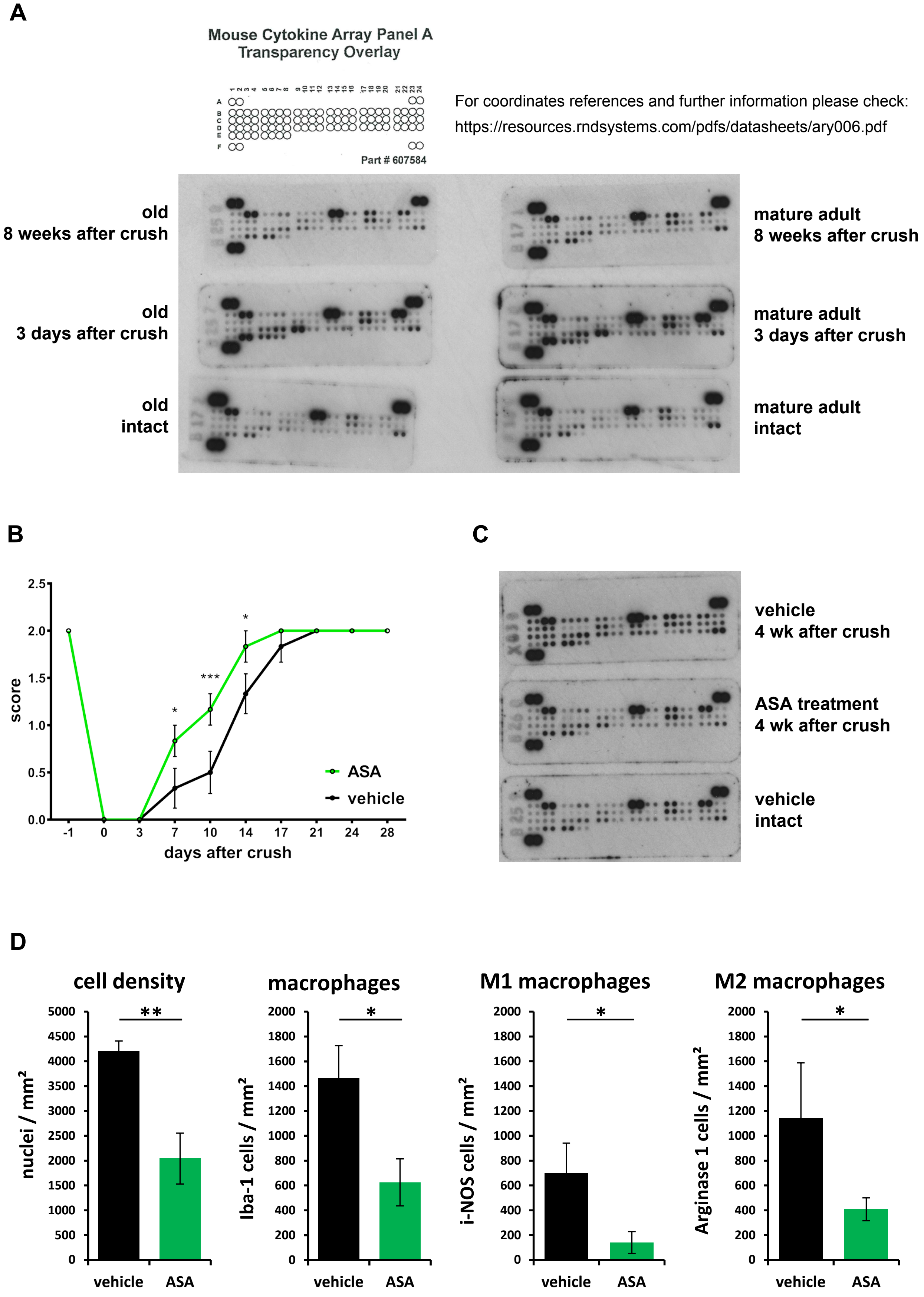
A
Mouse Cytokine Array Panel A
Transparency Overlay
Part # 607584
For coordinates references and further information please check:
https://resources.rndsystems.com/pdfs/datasheets/ary006.pdf
old
8 weeks after crush
old
3 days after crush
old
intact
mature adult
8 weeks after crush
mature adult
3 days after crush
mature adult
intact
B
score
days after crush
ASA
vehicle
C
vehicle
4 wk after crush
ASA treatment
4 wk after crush
vehicle
intact
D
cell density
nuclei / mm²
macrophages
Iba-1 cells / mm²
M1 macrophages
i-NOS cells / mm²
M2 macrophages
Arginase 1 cells / mm²
vehicle
ASA

Figure S3

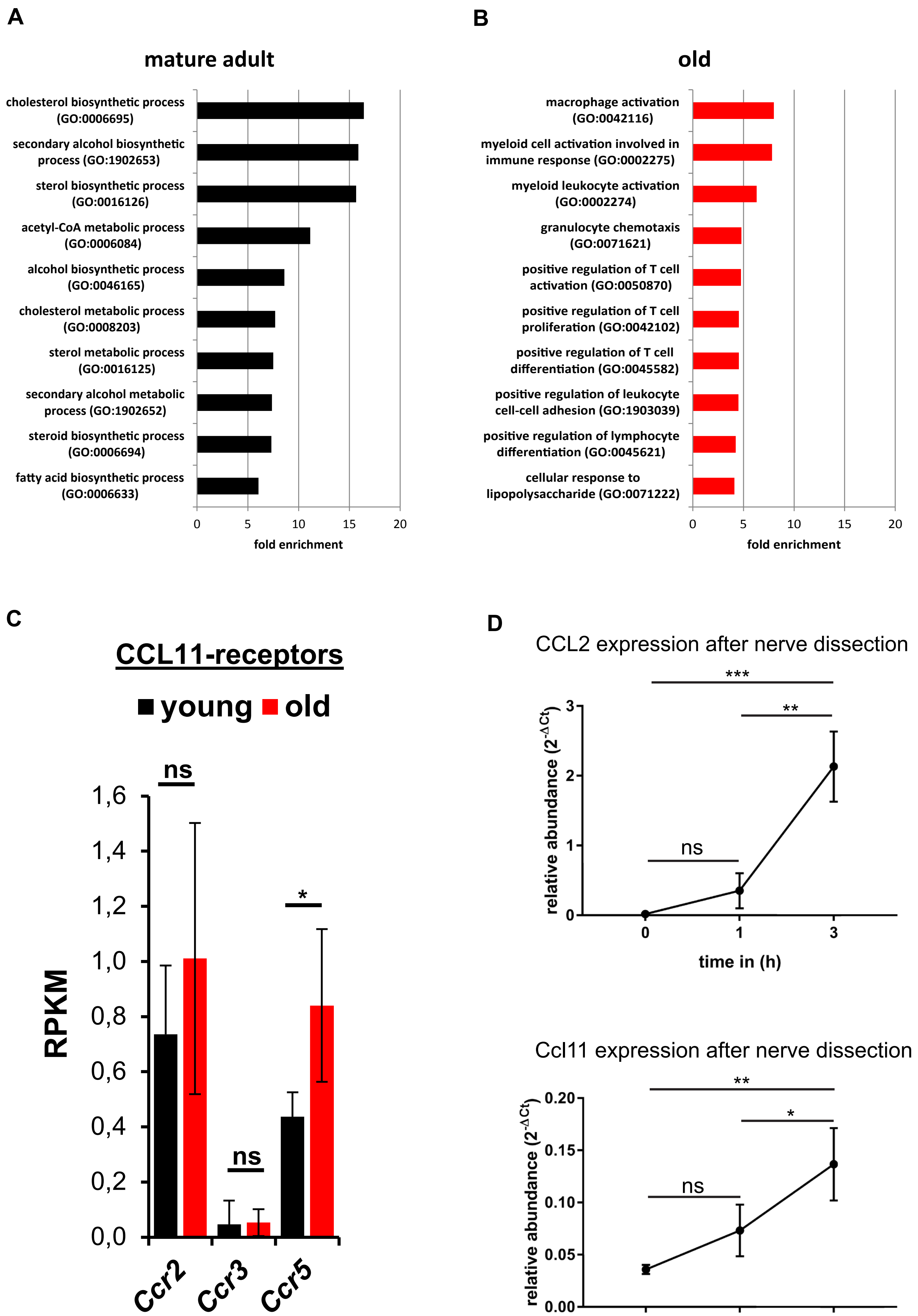
A
mature adult
cholesterol biosynthetic process (GO:0006695)
secondary alcohol biosynthetic process (GO:1902653)
sterol biosynthetic process (GO:0016126)
acetyl-CoA metabolic process (GO:0006084)
alcohol biosynthetic process (GO:0046165)
cholesterol metabolic process (GO:0008203)
sterol metabolic process (GO:0016125)
secondary alcohol metabolic process (GO:1902652)
steroid biosynthetic process (GO:0006694)
fatty acid biosynthetic process (GO:0006633)
0 5 10 15 20
fold enrichment
B
old
macrophage activation (GO:0042116)
myeloid cell activation involved in immune response (GO:0002275)
myeloid leukocyte activation (GO:0002274)
granulocyte chemotaxis (GO:0071621)
positive regulation of T cell activation (GO:0050870)
positive regulation of T cell proliferation (GO:0042102)
positive regulation of T cell differentiation (GO:0045582)
positive regulation of leukocyte cell-cell adhesion (GO:1903039)
positive regulation of lymphocyte differentiation (GO:0045621)
cellular response to lipopolysaccharide (GO:0071222)
0 5 10 15 20
fold enrichment
C
CCL11-receptors
young old
RPKM
1,6 1,4 1,2 1,0 0,8 0,6 0,4 0,2 0,0
ns
ns
*
Ccr2 Ccr3 Ccr5
D
CCL2 expression after nerve dissection
relative abundance (2^-ΔCt)
3 2 1 0
***
**
ns
0 1 3
time in (h)
Ccl11 expression after nerve dissection
relative abundance (2^-ΔCt)
0.20 0.15 0.10 0.05 0.00
**
*
ns
0 1 3
time in (h)

Figure S4

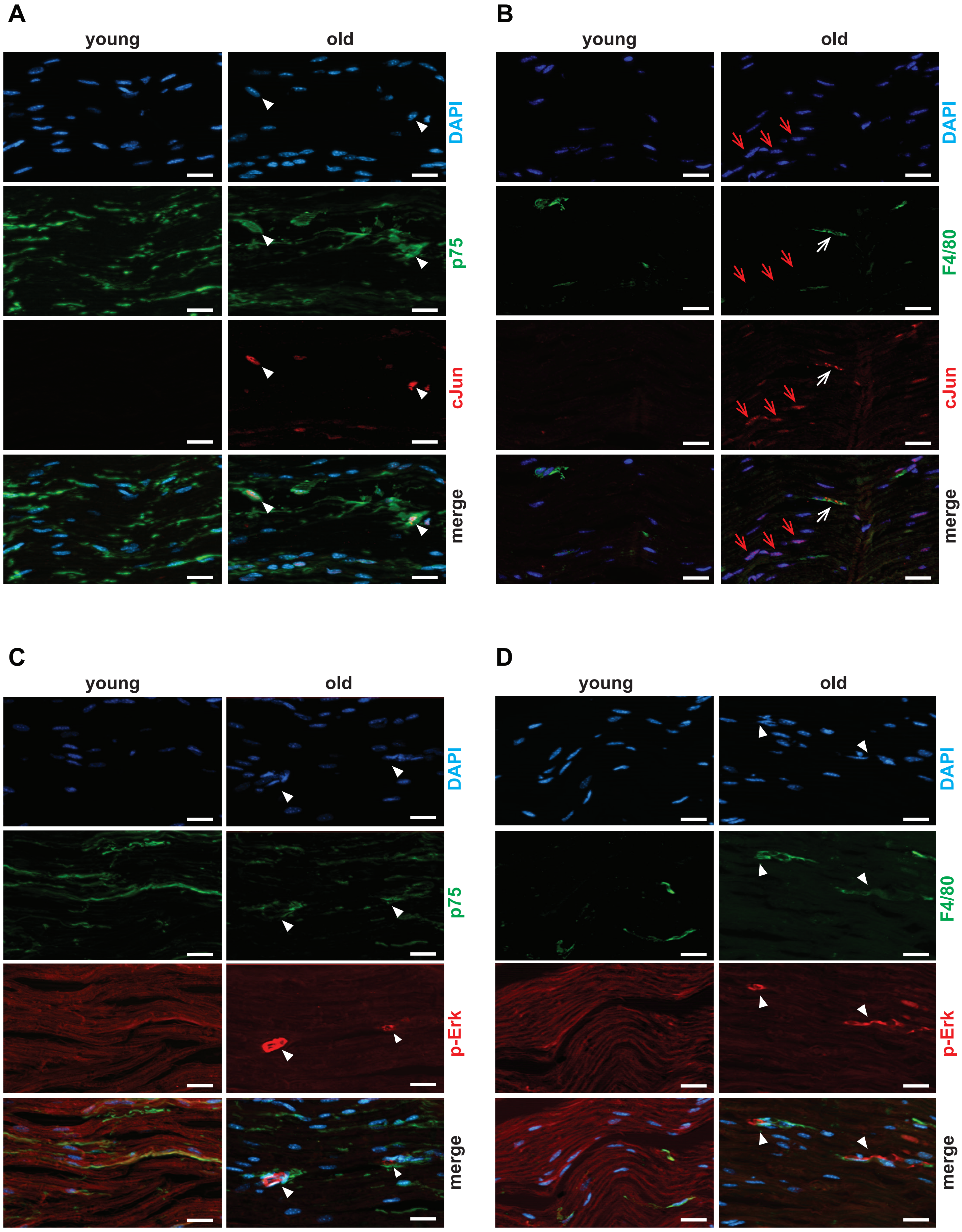
A
young
old
DAPI
p75
cJun
merge
B
young
old
DAPI
F4/80
cJun
merge
C
young
old
DAPI
p75
p-Erk
merge
D
young
old
DAPI
F4/80
p-Erk
merge

Supporting figure legends

Figure S1

Quantifications of axon density (**A**), average axon diameter (**B**), myelin thickness (**C**) and g-ratio (**D**) of sciatic nerves 4 weeks after crush injury (lesion side) and intact contralateral nerves (control side) from mature adult (3-6 months) and old mice (18-20 months). n = 4 mature adult mice and n = 5 old mice, between 156 and 448 axons plus myelin sheath were measured per sciatic nerve. *** $p < 0.001$ by Wilcoxon rank sum test (U-test).

Figure S2

(**A**) Dot blots of cytokine detection array for comparison of mature adult and old mice sciatic nerve lysates without crush and 3 days or 8 weeks after crush. Assay overlay is shown as provided by the manufacturer, hyperlink to assay layout is indicated as well. (**B**) Toe-spread test for motor reinnervation of 'old' mice treated with ASA (10 mg/kg body weight) or vehicle (PBS) following unilateral sciatic nerve crush. n = 6 mice per cohort, mean ± SEM. * $p < 0.05$ by two-way ANOVA with Holm-Sidăk multiple comparisons test. (**C**) Cytokine detection array dot blots for comparison of sciatic nerve lysates from uninjured or injured old mice 4 weeks after crush injury with or without ASA treatment. (**D**) Quantification of cell density, overall macrophage number, M1 and M2 macrophage populations in the crush area of sciatic nerves of 'old' mice 4 weeks after crush treated with ASA or vehicle; mean ± SD. n = 3 biological replicates. * $p < 0.05$, ** $p < 0.01$ in unpaired, two-tailed t-test.

Figure S3

The ten most enriched Gene Ontology (GO) groups of the differentially expressed genes in RNA-Sequencing analysis of intact sciatic nerves from mature adult (**A**) and old mice (**B**) calculated by PANTHER Enrichment analysis (www.geneontology.org) for biological processes with Bonferroni correction for multiple testing. Thereby 769 of the 1093 genes upregulated in sciatic nerves of mature adult mice were mapped and 877 of the 1230 genes upregulated in sciatic nerves of old mice, respectively. (**C**) RPKMs of the CCL11 receptors CCR2, CCR3, CCR5 calculated from RNA-Sequencing data of sciatic nerves isolated from 6 young and 6 old mice. Significant differences were determined by multiple t-test with Holm-Sidăk correction and illustrated by ** $p < 0.01$, mean ± SD. (**D**) Sciatic nerve explant experiment. RNA of sciatic nerves collected from n = 3 C57BL/6 mice (3-5 month old) was either immediately isolated (for 0 h time point) or kept in DMEM medium, supplemented with 1% heat inactivated fetal calf serum (FCS), 100 U/mL penicillin and 100 µg/mL streptomycin at 37°C for 1 h or 3 h followed by RNA isolation. Expression of MCP-1 (*Ccl2*) and CCL11 (*Ccl11*) was measured by real-time PCR. n = 3 biological replicates * $p < 0.05$, ** $p < 0.01$, *** $p < 0.001$ in one-way ANOVA + Tukey's multiple comparisons test.

Figure S4

Immunostained longitudinal sections of intact sciatic nerves from young and old C57BL/6 mice. (**A**) Co-staining of DAPI, p75 (marker for undifferentiated Schwann cells) and cJun, which are co-localized in old nerves (white arrowheads); scale bar: 50µm. (**B**) Co-staining of DAPI, F4/80 (marker for macrophages) and cJun. In old nerves cJun is rarely co-localized with F4/80 (white arrows) and if so, it is not nuclear; scale bar: 50µm. Nuclear cJun shows no co-localization with F4/80 (red arrows). (**C**) Co-staining of DAPI, p75 (marker for undifferentiated Schwann cells) and phospho-Erk, which are clustered in old nerves (white arrowheads); scale bar: 50µm. (**D**) Co-staining of DAPI, F4/80 (marker for macrophages) and phospho-Erk, which are also clustered in old nerves (white arrowheads); scale bar: 50µm.

Supporting experimental procedures

Experimental animals

All animal procedures were approved by the local authorities (Thüringer Landesamt für Verbraucherschutz, Germany) and conformed to international guidelines on ethical use of animals. Animals had free access to food and water and were housed under constant temperature and humidity conditions on a 12/12-h light/dark cycle. Old mice (20 months) as well as most of the young and mature adult mice (3-6 months) were purchased from Janvier Lab (Le Genest-Saint-Isle, France), remaining mice were bred in-house. All animals were on a C57BL/6J background.

Sciatic nerve crush injury

For unilateral injuries of sciatic nerves mice were anaesthetized with isoflurane in oxygen, fur was removed with an electric razor (Aesculap ISIS, B. Braun AG, Melsungen Germany) and skin incised minimally. The biceps femoris muscle was separated to reveal the sciatic nerve. Using a smooth hemostatic forceps (Fine Science Tools GmbH, Heidelberg, Germany) GmbH, Heidelberg, Germany) the nerve was crushed mid-thigh. Muscle tissue was sutured using non-absorbable surgical suture material (Catgut GmbH, Markneukirchen, Germany) and skin was closed by suturing or with an AutoClip System (FST).

Drug treatment

Application of ASA (Sigma-Aldrich, St. Louis, MO, USA) was performed as previously described (Schulz *et al.* 2016). ASA was dissolved in DMSO and diluted in phosphate-buffered saline (PBS) at a final concentration of

1 mg/ml. For systemic application, mice were injected intraperitoneally with 10 mg ASA per kg body weight for four weeks, beginning on the third day after crush injury and every second day thereafter. PBS without ASA served as vehicle control. Recombinant murine Eotaxin/CCL11 (PeproTech, Hamburg, Germany) was diluted in PBS at a final concentration of 1 µg/ml. For systemic application, mice were injected intraperitoneally every third to fourth day with 10 µg CCL11 per kg body weight, starting from one week before crush injury until four weeks after crush injury. PBS without CCL11 served as vehicle control.

Single frame motion analysis (SFMA)

To evaluate locomotor function by SFMA mice were accustomed to beam-walking before walking voluntarily from end to end on a horizontal beam. Rear views of walking trials were captured by a high definition video camera once prior and at indicated time-points after surgery. Video sequences were examined with VirtualDub 1.10.4 (GNU General Public License) and foot-base angles measured in selected frames with ImageJ v1.47t to serve as marker for reinnervation.

Toe-spread test

To test motor reinnervation, mice were placed on a transparent elevated walking beam. Once accustomed, mice walked voluntarily, and spreading of toes was recorded by a high definition video camera. Selected frames were analyzed in VirtualDub 1.10.4 (GNU General Public License) and scored according to Fig. 1d.

Semmes-Weinstein monofilament test

To investigate the sensory threshold of perception, animals were placed on a grid, accustomed and examined with Baseline® Tactile Semmes-Weinstein monofilaments (Fabrication Enterprises Inc., White Plains, NY, USA). Scoring was based on the threshold force of the most flexible filament still evoking a lifting of the paw: score 0 – no response; 1 – 300 g; 2 – 4 g; 3 – 2 g; 4 – 0.4 g; 5 – 0.07 g.

Electrophysiology

Mice were anaesthetized using isoflurane/O_2 inhalation. Hind limb fur was removed; needle electrodes were used to stimulate the left intact and right lesioned sciatic nerve *in-situ,* at a proximal and distal stimulation site separated by 12 mm. The neuromuscular response from the gastrocnemius muscle was recorded using a sensing ring electrode. A reference electrode was placed further distal. Nerve conduction velocity (NCV) and compound motor action potential (CMAP) were quantified from averaged response curves.

Immunohistochemistry

Paraffin-embedded sections of sciatic nerves were deparaffinized, rehydrated, boiled in 10 mM sodium citrate buffer for epitope retrieval and permeabilized with 0.5% Triton X-100. Afterwards, sections were incubated for at least 2 h in blocking buffer (0.2% fish skin gelatin, 1% goat serum and 1% donkey serum in PBS), followed by overnight incubation at 4 °C with primary antibody in blocking buffer. After multiple washing steps sections were incubated with secondary antibody at room temperature for 2 h. The

used primary and secondary antibodies are listed below. Finally, specimens were washed in PBS, counterstained with 1 µg/ml DAPI (Sigma-Aldrich, St. Louis, MO, USA) and embedded with Shandon™ Immu-Mount™ (Thermo Fisher Scientific Inc., Waltham, MA, USA). For phospho-ERK1/2 stainings, the Tyramide amplification kit (Thermo Fisher Scientific Inc., Waltham, MA, USA) was used. Sections were imaged on an AxioImager 2 microscope with ZEN2 software (both derived from Carl Zeiss GmbH, Jena, Germany).

List of primary and secondary antibodies for IHC

	**antibody name**	**order ID**	**manufacturer**
**primary**	P0	ab39375	Abcam, Cambridge, UK
	p75NTR	AB1554	Merck, Darmstadt, Germany
	Iba1	019-19741	WAKO, Richmond, VA, USA
	pan-axonal neurofilament	SMI312	BioLegend, San Diego, CA, USA
	iNOS	610329	BD Bioscience, Franklin Lakes, NJ, USA
	Arginase 1	sc-20150	Santa Cruz Biotech, Dallas, TX, USA
	phospho-ERK1/2	#4370	Cell Signaling, Danvers, MA, USA
**secondary**	Alexa 488, donkey anti-mouse	A21202	Thermo Fisher, Waltham, MA, USA
	Alexa 546, donkey anti-rabbit	A10040	
	Alexa 647, goat anti-chicken	ab150171	Abcam, Cambridge, UK

Morphometric and ultrastructural analysis of mouse sciatic nerves

Analysis of total axon number, axon diameter and myelination thickness was conducted on semi-thin sections of sciatic nerves, isolated from transcardially perfused mice. Mice were perfused with a solution containing 2 % paraformaldehyde and 2.5 % glutaraldehyde in 0.1 M cacodylate buffer (pH 7.4). Sections obtained from the distal part of the sciatic nerve were post-fixed for 1 h and kept in a fixative containing 3% sucrose. From each nerve 1 µm thick cross-sections were cut along the proximo-distal axis and distal segments were used about 1 mm away from the crushed side. Five sections

were used and random images of the toluidine blue-stained, semi-thin, cross-sections were taken using an Axioskop 2 MOT (Carl Zeiss GmbH, Jena, Germany), equipped with a 100× immersion oil objective and an Olympus XC50 digital camera (Olympus, Tokio, Japan). Standardized settings for camera sensitivity, resolution (2,576 × 1,932 pixels) and brightness of illumination were used for all micrographs. Image analysis was conducted with ImageJ v1.48u. RGB color images converted to greyscale, adjusted for contrast and axons and myelin were encircled using the hand tool. Axon and myelin were circumscribed manually using the freehand selection tool. Based on the measured areas, thicknesses of axons, thicknesses of myelin sheaths and g-ratios were calculated. At least 150 axons were evaluated per section. Statistics and graphs were made in R statistics.

Immunoblotting

Following SDS-PAGE, proteins were transferred to Roti®-NC nitrocellulose membranes (Carl Roth GmbH, Karlsruhe, Germany), which were incubated in blocking buffer containing 5% dried milk (Saliter, Obergünzburg, Germany) and 10% Roti-Block (Carl Roth) at room temperature for at least 1 h, followed by incubation with primary antibodies in blocking buffer overnight at 4 °C. Subsequently, membranes were washed three times with TBS-T buffer and incubated with secondary antibody in blocking buffer at room temperature for 1 h. The used primary and secondary antibodies are listed below. Membranes were washed three times with TBS-T, followed by development and visualization with Amersham ECL Western Blotting Detection Reagent (GE Healthcare Europe GmbH, Freiburg, Germany).

List of primary and secondary antibodies for Immunoblot

	antibody name	order ID	manufacturer
primary	GAPDH (6C5)	sc-32233	Santa Cruz Biotech, Dallas, TX, USA
	Erk1/2 (K-23)	sc-94/sc-153	
	Periaxin (G-5)	sc-515672	
	Egr-2	sc-293195	
	cJun	#9165	Cell Signaling, Danvers, MA, USA
	phospho-Erk1/2	#4370	
	Iba1	019-19741	WAKO, Richmond, VA, USA
	MBP	MAB384-1ML	Merck, Darmstadt, Germany
secondary	HRP-coupled anti-mouse	P0447	DAKO, Glostrup, Denmark
	HRP-coupled anti-rabbit	P0448	
	HRP-coupled anti-chicken	sc-2428	Santa Cruz Biotech, Dallas, TX, USA

RNA-Sequencing

Following sciatic nerve isolation both sciatic nerves per mouse were homogenized together in peqGOLD TriFast™ (VWR Peqlab, Darmstadt, Germany), total RNA was isolated by further purifying the aqueous phase with the PureLink RNA Mini Kit (Thermo Fisher Scientific Inc., Waltham, MA, USA) and RNA quantity was determined spectrophotometrically using a NanoDrop 2000 (Thermo Fisher Scientific Inc., Waltham, MA, USA). RNA quality was analyzed by Bioanalyzer 2100, using the RNA Nano Kit (both derived from Agilent Technologies Inc., Santa Clara, CA, USA). A RNA integrity number ≥7 was considered to be of good quality for RNA-seq. Approximately, 100 ng of total RNA was used for library preparation, using TruSeq RNA Sample Prep Kit v2 (Illumina Inc., San Diego, CA, USA). Library preparation and sequencing was conducted using Illumina's next-generation deep-sequencing platform. Libraries were sequenced using an Illumina HiSeq2500 in high-output 50-bp single-read mode, in pools of 5 per lane. Sequencing chemistry v3 was used. Read data were extracted in FASTQ

format using the Illumina-supported tool bcl2fastq v1.8.4. To detect differentially expressed genes (DEGs), the reads of all samples were mapped with TopHat2.0.6 to the mouse genome (mm10) with the ensemble gene annotation (release 85). For each annotated gene, reads that mapped uniquely to one genomic position were counted with FeatureCounts v1.4.3. Raw gene counts were used for gene-specific Z-score analysis. Additionally, the raw counts per gene of all 12 samples were analyzed with DESeq2 for differential expression. The p-value for each gene was calculated using the Wald significance test. The resulting p-values were adjusted for multiple testing with Benjamini & Hochberg correction (FDR) and the Log2-fold-changes of each expressed gene were calculated between the two pairs of sample groups. Genes with an adjusted p-value < 0.05 and an absolute Log2-fold-change > 0.5 were considered significantly differentially expressed. Finally the differentially expressed genes underwent a PANTHER Enrichment analysis (www.geneontology.org) for biological processes with Bonferroni correction for multiple testing.

DRG co-cultures

Dorsal root ganglia (DRGs) were dissected from in-house-bred C57BL/6J embryos (E13.5). DRGs were placed on Poly-L-Lysine/Laminine/Matrigel - coated coverslips (all Sigma-Aldrich, St. Louis, MO, USA) and cultivated for six days in cultivation medium (MEM (Thermo Fisher Scientific Inc., Waltham, MA, USA), Primocin (1:500, InvivoGen, Toulouse, France), 10% FCS (Thermo Fisher), 50ng/ml NGF (Sigma-Aldrich), 2 µM Forskolin (Merck-Millipore, Darmstadt, Germany), 10 ng/ml rhNRG1 (Peprotech, Hamburg, Germany), N2 Supplement (1:100, Thermo Fisher)). Afterwards, co-cultures

were cultivated for eight days in myelination medium (cultivation medium + 20 µM Forskolin + 50 ng/ml rhNRG1 + 50 µg/ml ascorbic acid (Sigma-Aldrich, St. Louis, MO, USA)). During the whole cultivation/myelination co-cultures were stimulated with CCL11 (100 ng/ml (Peprotech) + 0,1 % BSA in PBS) or vehicle (0,1 % BSA in PBS). After cultivation/myelination co-cultures were fixed with 4% PFA in PBS for 20 mins, washed several times with PBS and then blocked and stained with MBP (NB600-717; Novus Biologicals, Littleton, CO, USA) and neurofilament H (171102, Synaptic Systems, Göttingen, Germany) antibodies in blocking buffer containing 10% FCS, 5% Sucrose, 2% BSA and 0.3% Triton X-100 in PBS (all derived from Carl Roth GmbH, Karlsruhe, Germany).

Real-time PCR

RNA from mouse Schwann cells was purified with an RNeasy Mini Kit (Qiagen GmbH, Hilden, Germany) according to manufacturer's instructions. RNA from tissue was isolated using peqGOLD TriFast™ (VWR Peqlab, Darmstadt, Germany) until ethanol precipitation step followed by RNA purification using the PureLink RNA Mini Kit (AMBION GmbH, Kaufungen, Germany). RNA was eluted and analyzed using a NanoDrop 2000 device (Thermo Fisher Scientific Inc., Waltham, MA, USA). Afterwards, RNA was transcribed to cDNA using EvoScript Universal cDNA Master (Roche Diagnostics GmbH, Mannheim, Germany). Finally, gene expression was analyzed by real-time PCR using the A600A Go Taq® qPCR Master Mix (Promega, Madison, WI, USA) in a LightCycler® 480 (Roche Diagnostics GmbH, Mannheim, Germany). The used primer pairs are listed below.

List of primer pairs for real-time PCR

	gene	direction	primer sequences (5' → 3')
genes of interest	*MCP1*	forward<br>reverse	TTAAAAACCTGGATCGGAACCAA<br>GCATTAGCTTCAGATTTACGGGT
	CCL11	forward<br>reverse	GAATCACCAACAACAGATGCAC<br>ATCCTGGACCCACTTCTTCTT
	MBP	forward<br>reverse	TCACAGCGATCCAAGTACCTG<br>CCCCTGTCACCGCTAAAGAA
	MPZ	forward<br>reverse	CGGACAGGGAAATCTATGGTGC<br>TGGTAGCGCCAGGTAAAAGAG
	CDH1	forward<br>reverse	CAGGTCTCCTCATGGCTTTGC<br>CTTCCGAAAAGAAGGCTGTCC
	EGR2	forward<br>reverse	ACCTCCTTCCTACCCATCCC<br>ACAGGGAAACGGCTTTCGAT
	PRX	forward<br>reverse	GGCGGAGTTGGTGGAGATTA<br>AAAGAACACACGGGCACTCA
housekeep genes	*GAPDH*	forward<br>reverse	AGGTCGGTGTGAACGGATTTG<br>TGTAGACCATGTAGTTGAGGTCA
	RN18S	forward<br>reverse	CCAGAGCGAAAGCATTTGCC<br>GCATTGCCAGTCGGCATCGT
	PPIA	forward<br>reverse	GCAAGGATGGCAAGGATTGA<br>AGCAATTCTGCCTGGATAGC

Statistical analysis

Statistical analysis was performed using Prism 7 (GraphPad Software Inc., La Jolla, CA, USA). Dependent on the data (i) unpaired, two-tailed t-test, (ii) one-way ANOVA with Tukey post-hoc test, (iii) two-way ANOVA with Holm-Sidăk or Tukey's post-hoc test, (iv) linear mixed models with Tukey's post-hoc test or (v) Wilcoxon rank sum test (U-test) were used for analysis. A confidence interval of 95 % was applied in all cases and the p value calculated. Significant differences are indicated by asterisks: * ($p < 0.05$), ** ($p < 0.01$), *** ($p < 0.001$). All values are presented as means ± SD or SEM.
